# 

**Published:** 1905-12

**Authors:** 


### INDEX TO VOLUME XXVI.

### A

A consideration of the occlusal balance, 784
Allan, Charles F., M.D., Prophylaxis: its various phases in relation to conservation of the teeth, 120
A new porcelain for prosthetic purposes, 22
Angle, Edward H., M.D., D.D.S., Some studies in occlusion, 165
An interesting case, 382
Ankylostomiasis or urcinariasis, 810
A note on keramic fillings, 175
A practical way to improve dental college instruction, 648
A report of an unusual case of orthodontia, 511
A résumé of the study of saliva, 797
A simple, efficient crown for the posterior teeth, 454
A system for the surgical correction of harelip and cleft palate, 417

### B

Baker, Lawrence W., D.M.D., A report of an unusual case of orthodontia, 511
BIBLIOGRAPHY:
- A Practical Treatise on Artificial Crown, Bridge and Porcelain Work. By George Evans, 853
- Anæsthesia in Dental Surgery. By Thomas D. Luke, M.B., F.R.C.S.E., 409
- A Manual of Mechanical Dentistry and Metallurgy. By Geo. W. Warren, A.M., D.D.S., 558
- A Text-Book of Physiology, Normal and Pathological. By Winfield S. Hall, Ph.D., M.D. (Leipzig), 849
- Gray's Anatomy (new ed.), 557
- Henry's Appointment-Register-Day-Book for Dentists', 558
- Long's Dental Materia Medica and Therapeutics. By Eli H. Long, M.D., 852
- Manual of Chemistry. By W. Simon, Ph.D., M.D., 851
- Notes on Dental Porcelain. By V. Walter Gilbert, D.D.S., 555
- Notes on X-Light. By William Rollins, 139
- Oral Pathology and Therapeutics. By Elgin MaWhinney, D.D.S., 331
- Orthodontia and Orthopædia of the Face. By Victor Hugo Jackson, M.D., D.D.S., 73
- The American Text-Book of Operative Dentistry. By Edward C. Kirk, D.D.S., 847
- The Physicians' Visiting List for 1905, 78
- The Physicians' Visiting List for 1906, 854

Boenning, Henry C., M.D., Syphilis of the mouth, 169

Bogue, E. A., M.D., D.D.S,, The influence on development, of arranging irregularly placed teeth into normal positions, 761
Brigham, Dr. Walter I., Burnished gold fillings in soft cement, 803
Brown, Geo. V. I., A.B., D.D.S., M.D., C.M., A system for the surgical correction of harelip and cleft palate, 417
Brown, Geo. V. I., A.B., D.D.S., M.D., C.M., The vital significance of oral disease, 281
Brush, Dr. F. C., The idle moments of busy men, 528
Building, baking, and finishing an inlay, 584
Burnished gold fillings in soft cement, 803
Byram, Dr. J. Q., The rationale of the porcelain inlay, 497

### C

Carleton, Harry P., D.D.S., The dentistry of to-morrow, 716
Changes in salivary secretion, affected by systemic disease, 106
Chittenden, Charles C., D.D.S., What will probably be the dental educational standard for the coming decade? 577
Cleansing teeth, as the subject appears to the writer, 525
Conditions of failure, 358
Crothers, T. D., M.D., Some effects of inebriety on the teeth and jaws, 633
Cryer, M. H., M.D., D.D.S., Retarded eruption of the teeth: their liberation or extraction, 1
Current News:
A free dental service at the homes of the sick poor, 152
American Dental Society of Europe, 216
American Medical Association—Section on Stomatology, 339
American Society of Orthodontists, 858
California State Dental Association, 280
Clinic Section of the National Association, 494
Colorado State Dental Association, 567
Description of nude body of murdered woman found on Cutler Mountain, December 17, 1904, 150
Fargo District Dental Society, 496
"F.D.I." International Dental Federation, 344, 416, 496
First annual clinic of the Fraternal Dental Society of St. Louis, 632, 759
Golden anniversary at New Orleans, 152
Graduates of the Kansas City Dental College, 495
Joint Meeting of the Southern Branch of the National Dental Association and Tennessee Dental Association, 148
Harvard Dental Alumni Association, 566
Illinois State Dental Society, 568
Iowa State Dental Society, 280
Kentucky State Dental Association, 149
Lebanon Valley Dental Association, 344
Lewis and Clark Dental Congress, 412, 486
Maine Dental Society, 495
Minnesota State Board of Dental Examiners, 341

Current News:
Mississippi Dental Association, 649
Mississippi Valley Medical Association, 695
Missouri State Dental Association, 343, 567
National Dental Association clinic, 338
National Dental Association, 337, 415
National Association of Dental Faculties, 337, 696
National Dental Association, Section II, 493
New Jersey State Dental Society, 341
Northern Ohio Dental Association, 341
Offer of prizes by The New York Institute of Stomatology, 565
Officers of the Lewis and Clark Dental Congress, 215
Officers of the National Association of Dental Examiners, 695
Oklahoma Dental Association, 343
Pennsylvania Association of Dental Surgeons, 80
Pennsylvania State Board of Dental Examiners, 280
South Dakota State Board of Dental Examiners, 344, 416, 496, 858
The Fraternal Dental Society of St. Louis, Mo., 152
XV Congrès International de Médecine, Lisbonne, 19–26 avril, 1906, 755

D

Davenport, Dr. S. E., Some partial impressions, 645
Dental education from the view of experience, 372
Distal and mesial occlusion: some causes and some results, 153
Dowski, Samuel, D.D.S., A résumé of the study of saliva, 797
Domestic Correspondence:
Correction, 214
Correspondence on orthodontia, 483
Letter from Dr. Talbot, 142
Make-up of dental profession in Great Britain, 559
National Association of Dental Examiners, 277
Teeth receding in vulcanization, 629
The oath of Hippocrates, 628
Dr. J. N. Farrar's appliances, 294

E

Editorial:
Acknowledgment, 139
British Dental Association, 72
Correction, 330
Dr. Charles C. Chittenden, 846
Has the lecture a future in education? 551
Hasty conclusions, 480
Indentification through the teeth, 138
Lewis and Clark Dental Congress, Portland, Oregon, 212
Notice to all interested, 839
Notice to exchanges, 846
Past, present, and future of mechanical dentistry, 687
Notice to exchanges, 846

EDITORIAL:
The cause of failure, 839
The conventions at Buffalo, 623
The *Dental Digest* hysterical, 275
The *Dental Register* and quinine, 269
The last word, 842
The faddist in dentistry, 135
The fifth year of the century, 69
The New York Institute of Stomatology—offer of prizes, 554
The oath of Hippocrates, 627
The position of dentistry in relation to medicine, 750
What of the harvest? 406
Whither are we drifting? 327
Electrolysis for the treatment of peridental inflammations, 32
Errors, mistakes, and failures old and new, 639
Extension of preliminary education *versus* extension of the dental course, 569

F

Fillebrown, Thomas, M.D., D.M.D., Three cases in oral surgery, 726
Fossume, F. L., The matrix, 590
FOREIGN CORRESPONDENCE:
Personal explanation, 278

G

Gelston, William H., D.D.S., Plaster of Paris, 289
Gold plates having teeth attached with vulcanite, 365
Gonorrhœal ulcero-membranous stomatitis, 225
Goslee, Hart J., D.D.S., The rationale of pulp extirpation, 228

H

Hamilton, Dr. Harry F., The successes and failures in our daily work, 733
Hofheinz, R. H., D.D.S., Extension of preliminary education *versus* extension of the dental course, 569
Hopkins, Samuel A., M.D., D.D.S., Some recent observations in metabolism and their importance in dentistry, 345
Howe, Horace L., D.M.D., Distal and mesial occlusion: some causes and some results, 153
Howe, Horace L., D.M.D., The relation of thorough mouth treatment of food to the affections of the teeth, 367
Hurd, Lee Maidment, M.D., The radical treatment of the maxillary antrum, 159

I

Inaugural address, 706

J

Jenkins, Dr. N. S., A new porcelain for prosthetic purposes, 22

L

Lawshe, Allison R., D.D.S., A simple, efficient crown for the posterior teeth, 454

Lederer, William J., D.D.S., and Heinrich Stern, M.D., Changes in the salivary secretion, affected by systemic disease, 106

Leonard, T. M. Russell, L.R.C.P., L.R.C.S. (Ed.), Ankylostomiasis or urcinariasis, 810

Lewis, O. G. L., D.D.S., An interesting case, 382

M

Macpherson, Dr. A. H., Electrolysis for the treatment of peridental inflammations, 32

McManus, Dr. Charles, On the history of dentistry from the earliest times to the founding of the profession by Hayden and Harris; with special reference to the immediate publication of such a work in English, 826

McLaren, F. J., Gold plates having teeth attached with vulcanite, 365

Miller, Willoughby D., A.M., D.D.S., M.D., Sc.D., Inaugural address, 706

MISCELLANY:

- An adjustable indicator, 411
- An automatic cylinder for ethyl chloride, 336
- An everted rim tumbler, 336
- An automatic anæsthetic capsule-holder and breaker, 691
- An interesting fact in Hayden's life, 80
- A new property of aluminum, 564
- A novel head-rest, especially useful in tooth-extraction, 693
- Business and profession, 334
- Caustic soda for cleansing bridges and regulating apparatus, 631
- Caution: death from blood-poisoning, following local anæsthesia for tooth extraction, 630
- Dental examining glass, 336
- Dr. Endelman on stomatitis, 333
- Dr. Rudel's detachable wedge separator, 335
- Fountain spittoon, 336
- French dental college in Canada, 563
- New appliances advertised in the English dental journals—1904, 146
- New grip gags, 412
- Position of flask in vulcanizer, 564
- Solution of shellac in borax water for coating plaster casts, 564
- Teeth receding in vulcanization, 563
- To prevent bubbling of borax while soldering, 564
- Tulloch's alloy and mercury measures, 336
- The influence of the first and second dentition periods in the etiology of epilepsy, 692

O

OBITUARY:

- Durand, Dr. Frederic Frank, 854
- Goddard, Clark La Motte, A.B., D.D.S., A.M., 560
- Harriman, George B., D.D.S., 856
- Hutchinson, Samuel John, M.R.C.S., L.D.S. (Eng.), 78
- Resolutions of respect to Dr. Ellery C. Young, 562
- Resolutions of respect to Dr. George E. Nettleton, 754
- Wilson, Dr. Wm. N., 856

O'Brian, Dr. L. A., Building, baking, and finishing an inlay, 584
On the history of dentistry from the earliest times to the founding of the profession by Hayden and Harris; with special reference to the immediate publication of such a work in English, 826
ORIGINAL COMMUNICATIONS:
A consideration of the occlusal balance, 784
A new porcelain for prosthetic purposes, 22
An interesting case, 382
Ankylostomiasis or urcinariasis, 810
A practical way to improve dental college instruction, 648
A note on keramic fillings, 175
A report of an unusual case of orthodontia, 511
A résumé of the study of saliva, 797
A simple, efficient crown for the posterior teeth, 454
A system for the surgical correction of harelip and cleft palate, 417
Building, baking, and finishing an inlay, 584
Burnished gold fillings in soft cement, 803
Changes in the salivary secretion, affected by systemic disease, 106
Cleansing teeth as the subject appears to the writer, 525
Conditions of failure, 358
Dental education from the view of experience, 372
Distal and mesial occlusion; some causes and some results, 153
Dr. J. N. Farrar's appliances, 294
Electrolysis for the treatment of peridental inflammations, 32
Errors, mistakes, and failures, old and new, 639
Extension of preliminary education *versus* extension of the dental course, 569
Gold plates having teeth attached with vulcanite, 365
Gonorrhœal ulcero-membranous stomatitis, 225
Inaugural address, 706
On the history of dentistry from the earliest times to the founding of the profession by Hayden and Harris; with special reference to the immediate publication of such a work in English, 826
Plaster of Paris, 289
Porcelain inlays from the clinical standpoint— cavity preparation, 582
Preventive dentistry, 239
Prophylaxis: its various phases in relation to conservation of the teeth, 120
Retarded eruption of the teeth: their liberation or extraction, 1
Should the dental practitioner concern himself with the chemical examination of saliva or urine? 24
Solbrig's porcelain jacket crown, 652
Some effects of inebriety on the teeth and jaws, 633
Some partial impressions, 645
Some recent observations in metabolism and their importance in dentistry, 345
Some studies in occlusion, 165

ORIGINAL COMMUNICATIONS:
Study of the pithecanthropus erectus, or ape-man, 650
Surgical aspects of disturbed dentition of the third molars, 697
Syphilis of the mouth, 169
The dentistry of to-morrow, 716
The etiology, pathology, and treatment of troublesome tooth sockets 217
The first dental work published in the United States, 337
The idle moments of busy men, 528
The influence, on development, of arranging irregularly placed teeth into normal positions, 761
The matrix, 590
The packing of amalgam, with a review of some literature upon the subject, 515
The practical way to improve dental college instruction, 648
The radical treatment of the maxillary antrum, 159
The rationale of pulp extirpation, 228
The rationale of the porcelain inlay, 497
The relation of the dentist to the nose, throat, and ear specialist, 81
The relation of thorough mouth treatment of food to the affections of the teeth, 367
The successes and failures in our daily work, 733
The vital significance of oral disease, 281
Three cases in oral surgery, 726
What will probably be the dental educational standard for the coming decade? 577

P

Palmer, James G., Porcelain inlays from the clinical standpoint—cavity preparation, 582
Plaster of Paris, 289
Porcelain inlays from the clinical standpoint—cavity preparation, 582
Preventive dentistry, 239
Prophylaxis: its various phases in relation to conservation of the teeth, 120

R

REPORTS OF SOCIETY MEETINGS:
Academy of Stomatology, 203, 261, 320, 396, 467, 542, 597, 675
American Academy of Dental Science, 837
The New York Institute of Stomatology, 61, 127, 195, 315, 389, 460, 534, 595, 670, 832
Thirty-fifth annual session of the New Jersey State Dental Society, 617
Ninth annual meeting of the National Dental Association, 678
Retarded eruption of the teeth: their liberation or extraction, 1
REVIEWS OF DENTAL LITERATURE:
A brief review of the prevailing theories of immunity, 305
Actinomycosis of the mouth and face, with the report of ten cases, 299

REVIEWS OF DENTAL LITERATURE:
Anæsthesia, local, 260
An interesting case in practice, 667
Brief notes on pyorrhœa alveolaris in India, 246
Compressed air in dentistry, 310
Contribution to the pathology and treatment of tic douloureux of the face, 741
Further experience of cement and amalgam fillings 244
Hand sterilization, 531
Highest orthodontia: 1, Facial beauty; 2, Dental antagonism, 295, 383, 455
Parotid gland, 533
Pathogeny of osteomalacia or senile atrophy, 43
Professional emancipation and how it may be effected, 247
Researches in hæmophilia, 532
Six years' work in oral prophylaxis, 176
Some studies on the anatomy of the teeth and their practical operation, 590
The antimicrobic action of iodine, 657
The choice and use of medical literature, 35
Vulcanite crowns (*British Dental Journal*), 241
Why use low-carat solder on new dental work? 745
Rhein, M. L., M.D., D.D.S., Surgical aspects of disturbed dentition of the third molars, 697
Richards, George L., M.D., The relation of the dentist to the nose, throat, and ear specialist, 81
Rollins, William, A note on keramic fillings, 175

S

Schamberg, M. I., D.D.S., M.D., The etiology, pathology, and treatment of troublesome tooth sockets, 217
Should the dental practitioner concern himself with the chemical examination of saliva or urine, 24
Smith, H Carleton, Ph.D., Should the dental practitioner concern himself with the chemical examination of saliva or urine? 24
Solbrig's porcelain jacket crown, 652
Spaulding, John H., Solbrig's porcelain jacket crown, 652
Stern, Heinrich, M.D., and Lederer, William J., D.D.S., Changes in the salivary secretion, affected by systemic disease, 106
Strang, Dr. C. W., Errors, mistakes and failures, old and new, 639
Some effects of inebriety on the teeth and jaws, 633
Some partial impressions, 645
Some recent observations in metabolism and their importance in dentistry, 345
Some studies in occlusion, 165
Study of the pithecanthropus erectus, or ape-man, 650
Surgical aspects of disturbed dentition of the third molars, 697
Syphilis of the mouth, 169

### T

Talbot, Eugene S., M.S., D.D.S., M.D., LL.D., Study of the pithecanthropus erectus, or ape-man, 650

Talbot, Eugene S., M.S., D.D.S., M.D., LL.D., Gonorrhœal ulcero-membranous stomatitis, 225

The dentistry of to-morrow, 716

The etiology, pathology, and treatment of troublesome tooth sockets, 217

The first dental work published in the United States, 377

The idle moments of busy men, 528

The influence, on development, of arranging irregularly placed teeth into normal positions, 761

The matrix, 590

The packing of amalgam, with a review of some literature upon the subject, 515

The practical way to improve dental college instruction, 648

The radical treatment of the maxillary antrum, 159

The rationale of pulp extirpation, 228

The rationale of the porcelain inlay, 497

The relation of the dentist to the nose, throat, and ear specialist, 81

The relation of thorough mouth treatment of food to the affections of the teeth, 367

The successes and failures in our daily work, 733

The vital significance of oral disease, 281

Three cases in oral surgery, 726

Trueman, William H., D.D.S., The first dental work published in the United States, 377

Trueman, William H., D.D.S., A practical way to improve dental college instruction, 648

Truman, James, D.D.S., LL.D., Dental education from the view of experience, 372

### W

Wedelstaedt, E. K., Conditions of failure, 358

What will probably be the dental educational standard for the coming decade? 577

Wheeler, Herbert Locke, D.D.S., The packing of amalgam, with a review of some literature upon the subject, 515

Whitlock, Dr. W. M., Cleansing teeth, as the subject appears to the writer, 525

Wright, C.M., A.M., D.D.S., Preventive dentistry, 239

